# Scenting the Anosmic Cube: On the Use of Ambient Scent in the Context of the Art Gallery or Museum

**DOI:** 10.1177/2041669520966628

**Published:** 2020-11-20

**Authors:** Charles Spence

**Affiliations:** Crossmodal Research Laboratory, Department of Experimental Psychology, University of Oxford, Oxford, United Kingdom

**Keywords:** multisensory experience design, olfaction, art, congruency, museum

## Abstract

In recent years, there has been growing interest in the possibility of augmenting the visitor’s experience of the exhibits in various art galleries and museums by means of the delivery of a genuinely multisensory experience, one that engages more than just the visual sense. This kind of approach both holds the promise of increasing engagement while, at the same time, also helping to address, in some small way, issues around accessibility for the visually impaired visitor. One of the increasingly popular approaches to enhancing multisensory experience design involves the use of scents that have been chosen to match, or augment, the art or museum display in some way. The various different kinds of congruency between olfaction and vision that have been investigated by researchers and/or incorporated into art/museum displays already are reviewed. However, while the laboratory research does indeed appear to suggest that people’s experience of the paintings (or rather reproductions or photos of the works of art) may well be influenced by the presence of an ambient odour, the results are by no means guaranteed to be positive, either in terms of the emotional response while viewing the display or in terms of the viewer’s subsequent recall of their multisensory experience. As such, caution is advised for those who may be considering whether to augment their multisensory displays/exhibits with ambient scent.

Traditionally, art galleries and museum displays have appealed primarily to just one sense, namely vision. And while once upon a time the occasional visitors would sometimes apparently have been allowed to handle the exhibits (e.g., see Candlin, 2003, 2004, 2006, 2010; Classen, 2005, 2007, 2017), issues of conservation mean that such multisensory engagement with the exhibits on display has mostly been prevented in recent times (though see Howes, 2014). At the same time, however, many publically funded museums and art galleries are increasingly being expected to justify their value for money by demonstrating the public’s engagement with the displays and exhibitions on show (e.g., Bourgeon-Renault, 2000; Colbert, 2009, 2012; Gilmore & Rentschler, 2002; Harrison & Shaw, 2004). As a result, a growing number of venues are increasingly trying to engage their visitors’ senses more effectively to deliver multisensory experiences and not just merely a ‘feast for the eyes’ (Joy & Sherry, 2003).^1^ Indeed, one now finds a growing number of commentators talking about the multisensory museum (e.g., Classen, 2014, 2017; Howes, 2014; Levent & Pascual-Leone, 2014; see also Henshaw et al., 2018; Losche, 2006; Vega-Gómez et al., 2020). It is in this context, then, that ideas around multisensory experiential marketing have become increasingly relevant/important to the museum/gallery sector (e.g., Bourgeon-Renault et al., 2006; Gómez & van der Woude, 2013; McIntyre, 2009; Petkus, 2004; Yucelt, 2000).

The past few years or so has also seen “a rising tide of sensory experimentation in contemporary curatorial practice” (Howes, 2014, p. 259), along with an explosion of multisensory tasting events held in museums (such as the Musical Instrument Museum in Brussels, Belgium, http://mim.be/sonic-taste-0; Miraikan, The National Museum of Emerging Science and Innovation in Tokyo, https://www.miraikan.jst.go.jp/en/; the Museum aan de Stroom in Antwerp, https://www.mas.be/en/content/welcome; the Cosmos Coffee exhibition at the Deutsches Museum in Germany, https://www.deutsches-museum.de/en/exhibitions/special-exhibitions/cosmos-coffee/; and Museum Tinguely in Basel, Switzerland, https://www.tinguely.ch/en.html) that are both experimental and experiential in nature (e.g., Museum Tinguely, Basel, 2020; Reinoso Carvalho et al., 2016; Reinoso-Carvalho et al., 2019b, 2020; Spoerri, 2020; Wang et al, 2017).^2^

At the same time as they try to increase visitor numbers by engaging more of the latter’s senses, however, there is also a growing sense that the majority of museums and galleries need to do more to create engaging multisensory experiences for those visitors who may be visually impaired (European Blind Union, 2018). The introduction of non-visual elements into the experience, be it in an art gallery or museum, can help to address issues around enhanced accessibility for the blind and partially sighted (e.g., Davidson et al., 1991; European Blind Union, 2018; Graven et al., 2020). Relevant here, Spence (2020b) has drawn attention to the increasingly common strategy in book/manuscript exhibitions to introduce a variety of multisensory elements. So, for example, several recently exhibitions have chosen to emphasize the smell/feel of old manuscripts (Bembibre & Strlič, 2017; see also Bacci & Pavani, 2014; Classen, 2017; Classen & Howes, 2006; Levent & Pascual-Leone, 2014). There has been a conscious effort to go beyond the visual—that is, merely showing books and manuscripts behind protective glass display cases—in part, to address issues of accessibility (see also Howes, 2014). Stevenson (2014) also mentions a couple of other examples where scented elements have been introduced into museum tours for the visually impaired visitor, including the Vatican Museum, with linen shrouds smelling of myrrh and aloe, and at the Brooklyn Museum of Art that include the opportunity to smell components of particular pictures (also see Malvern, 2019).

Until recently, however, there has been little attempt to deliberately introduce scent into the sighted visitor’s experience of museums or art galleries (though see Castel, 2018). Jim Drobnick (2005) captures this absence of olfactory stimulation when talking about not just the “white cube” mentality when displaying art (first highlighted by Brian O’Doherty, 1976, 2009; see also Pursey & Lomas, 2018) but of the “anosmic cube” (see also el-Khoury, 2006). By introducing this phrase, Drobnick draws attention to the fact that most art galleries have no scent whatsoever nor, one might say, are they necessarily particularly amenable to the introduction of an olfactory element either.^3^ In fact, it could even be argued that the gallery/museum is perhaps just one of those public spaces that people expect to be scentless. According to Parsons (2009), those retail stores that normally do not have a scent need to be particularly careful when introducing an olfactory element to make sure that it is somehow associated with the type of store; otherwise, they may risk a negative response from their customer base. One might wonder at the outset here whether the same is likely to be true in the art gallery and/or museum context as well.

However, regardless of what the response of visitors might turn out to be, a growing number of art galleries and museums have started to scent the exhibit/exhibitions. For instance, Drobnick (2014) highlights the use of Indian incense at “The Arts of the Sikh Kingdoms” (1999) at the Victoria and Albert Museum in London and green tea and sandalwood sticks infusing the air at the Österrichische Galerie Belvedere collection (Egon Scheiele’s *Sunflowers I*, 1911, and Gustav Klimt’s *The Kiss*, 1908). Meanwhile, Stevenson (2014) lists 12 museums around the world that have used ambient odours to help create multisensory exhibits. And beyond these examples, the *Ultra Peau* exhibition at the Palais de Tokyo (France) in April 2006 took visitors on an interactive journey to “the land of a thousand-and-one sensations” with olfactory chambers, tactile walls, and so forth. “Visitors are exposed to sounds, colours, materials.” Meanwhile, a perfumer recreated the smells of a Greek trading station in the Ancient World as part of the *Amphora à la Mer* exhibition (Pulh et al., 2008).

While a few studies assessing the impact of introducing an olfactory element into the art gallery/museum have now been conducted, the majority of research to date has tended to be laboratory-based. What this review of the literature reveals is that while, in certain cases, the scent has been chosen to be (e.g., hedonically) congruent with the works on display, in other cases, it has been entirely unrelated. What is also apparent from reviewing the literature is that while museum displays have typically chosen to use (mostly synthetic) smells linked semantically to what can be seen (e.g., the smell of apple pie in a kitchen display), when it comes to scenting art, researchers have typically been more interested in the impact of hedonically congruent/mismatching scents instead. While the design of the various laboratory studies has sometimes stressed the link between a given scent and an associated picture, in other cases, it has been nothing more than an unrelated background ambient scent (either pleasant or unpleasant) in the space where the participants happen to be. In other words, the degree of connection, or correspondence, between the visual displays and the olfactory stimulation has varied widely from one study to the next.

In the laboratory setting, the research has typically revolved around the question of whether people’s hedonic and/or aesthetic response to the art is affected by the presence of ambient scent. By contrast, in the context of the museum, the focus has tended to be on lingering time, subsequent memory for the content of the displays, and the visitor’s intention to return. And while the majority of the published research suggests that people’s response to viewing reproductions or photos of artworks and/or museum displays is indeed influenced by the introduction of ambient scent, the change is by no mean always positive (see also Cirrincione et al., 2014, on this theme). In part, this may depend on quite what the association between the scent and the artworks and museum displays happens to be, and how exactly it is introduced and/or explained. Given the available evidence, caution is advised for those practitioners who may be considering whether to augment their multisensory displays and exhibits with ambient scent. Simply adding more senses to the visitor’s experience is by no means guaranteed to deliver a positive outcome, however that is defined.

The aim of this literature review, then, is to highlight these challenges/constraints in scenting the anosmic cube. The next section reviews the literature documenting how scent has been incorporated into a variety of art galleries and museums. Then, the Olfactory Modulation of Art: Laboratory Research section reviews the laboratory research concerning the impact of ambient scent on the visual perception of art. This is followed by a section that provides a number of possible explanations for why ambient scent might influence the perception of art/museum displays in the way it has been shown to do. This leads on briefly to the question of how, exactly, crossmodal congruency should be defined in the context of scenting the art gallery or museum (see Crossmodal Congruency: How Is It To Be Defined? section), before I summarize the findings and draw a number of key conclusions in the final section.

## Can You Smell the Art?

In recent years, a growing number of museums, and increasingly also art galleries, have started to introduce scent, either on a temporary or permanent basis. For instance, one such attempt to engage more of the visitors’ senses in the gallery setting (on a temporary basis) took place in 2019 in Paris. In this case, a number of top *noses* (i.e., perfume makers) were commissioned to create scents to match eight of the masterpieces from The Louvre’s permanent collection. According to the press coverage that appeared at the time (e.g., Bremner, 2019; Richardson, 2019; Thomas, 2019), the idea was to add a new sensory dimension to the visitor experience and, by so doing, *awaken all the senses.* The paintings chosen for this exercise included a couple of Ingres nudes, *The Valpinçon Bather* and *La Grande Odalisque*, Fragonard’s *The Bolt*, and a somewhat lesser known work by Thomas Gainsborough, entitled *Conversation in a Park*. Meanwhile, the sculptures included the *Venus de Milo*, goddess of love, and the *Winged Victory of Samothrace.*^4^

The challenge for the creative perfumers in this case was to try and evoke the selected works of art by means of the stimulation of the visitor’s nostrils. Reading about the event in the press, one could all too easily have come to the conclusion that the scent was actually infused into the relevant galleries where the featured artworks were on display. Sadly, however, this was not the case in this instance. The decision not to scent the galleries perhaps reflecting the fact that it can be challenging to constrain the diffusion of ambient scents through large open spaces (Drobnick, 2014; Keller, 2014), especially when there are lots of people milling around. What this means, in practice, is that a scent that has been chosen to complement, or match, a particular work of art, could all too easily end up “fragrancing” another work with which it might not be quite so congruent if situated nearby (e.g., in the same or adjacent gallery). In the case of The Louvre, curious visitors were simply invited to purchase the fragrances from the museum shop instead. One does, though, have to wonder quite how many people were actually tempted to pay for such an idiosyncratic olfactory experience.^5^ That said, the Louvre is by no means the first museum to offer an olfactory tie-in. For, according to Drobnick (2014), the Philadelphia Museum of Art apparently offered perfume to accompany their 2008 Frida Kahlo exhibition, while the Art Gallery of Ontario did the same for their Salvador Dali exhibition entitled Surreal Things in 2009. Note that a scented tie-in was also introduced in the early days of scented cinema (see Gilbert, 2008; Spence, 2020c).

### Welcome to the Tate Sensorium

A rather more intriguing attempt to incorporate scent into the visitor’s experience came from Flying Object, a London-based creative studio, and winner of the 2015 IK prize. This is an annual prize awarded for the innovative use of digital technology to engage the public with Tate Britain’s vast collection of British art (Davis, 2015). Four of the paintings from the collection were chosen for the *Tate Sensorium* exhibition from three different decades. Flying Object developed a multisensory experience around each work incorporating the latest in digital technology (including midair ultrahaptics, directional sound, and the movement-triggered release of scent; Vi et al., 2017). This experience was, though, limited with only four people being allowed in the one-room exhibition space at any one time (Davis, 2015). Nevertheless, the experience was a sellout, with a 100% capacity of 4,000 visitors over the 2 months of the exhibit (see Pursey & Lomas, 2018), perhaps hinting at the appeal of multisensory experience design more generally (Volpicelli, 2015). The installation was set up as an experiment with the visitors wearing wireless galvanic skin response wristbands to measure their responses to the multisensory exhibits. The suggestion was that the data so collected would provide relevant scientific insights (Davis, 2015), though 5 years on, I have yet to see any scientific publications emerging from this work.

Of particular relevance to the present review, two of the four works at the *Tate Sensorium* were accompanied by scents, and a third was to be viewed while tasting an aromatic, smoky, dark chocolate, thus presumably stimulating olfaction retronasally. For instance, in the case of Richard Hamilton’s (1964) *Interior II,* a Black-and-White full-bodied screen print portrait of actress Patricia Knight from the 1949 movie *Shockproof* dominates the scene. The smell of wood polish (Pledge) was introduced to reference the parquet flooring depicted in the scene. A bespoke carnation perfume was dispensed from another device to link to the scent of hair spray, often mentioned in the film itself. Finally, the smell of glue points to the use of collage in the creation of Hamilton’s work. The release of each scent was triggered by hidden movement detectors when the visitor moved around in front of the work (Pursey & Lomas, 2018). There was also an accompanying soundtrack, a kind of auditory collage, of elements shown in the scene, including the sound of heels on the floor, traffic through the window, and the scene depicted on the TV in the painting.

**Figure 1.**
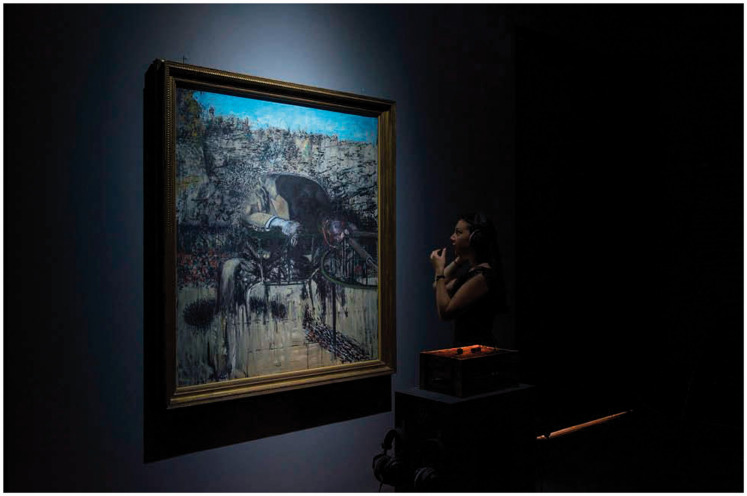
Installation Shot of the IK Prize: Tate Sensorium at Tate Britain With Francis Bacon’s Figure in a Landscape (1945). The viewer is wearing headphones and eating chocolate (photo courtesy of Tate Photography; as presented in Pursey & Lomas, 2018).

Meanwhile, David Bomberg’s *In the Hold* (c. 1913–1914), made up of a dizzying array of black and coloured triangles, was to be experienced while inhaling the contents of one of two triangular-shaped salt shakers. The suggestion was that the high-pitched scent in one *shaker* would bring out the blues in the scene, while the scent in the other would draw the viewer’s (smeller’s?) attention to the browns and ochers instead.^6^ Francis Bacon’s (1945) *Figure in a Landscape* was to be viewed while tasting a chocolate composed of edible charcoal, sea salt, burnt orange, cacao nibs, and smoky lapsang souchong tea designed, apparently, to bring out the painting’s dark nature while at the same time listening to an audio track over headphones that was composed of mechanized industrial sounds (see Figure 1). The fourth work in the *Tate Sensorium* exhibition, John Latham’s (1961) *Full Stop,* incorporated ultrahaptics and a soundscape, but no scent-sory element.

### Scenting the Museum

For decades now, ambient scents have been incorporated in various museum exhibits (e.g., Aggleton & Waskett, 1999; Stevenson, 2014; Walsh, 1992, p. 101) and entertainment locations, such as theme parks (Mack, 2014). There is even mention of trade shows being scented in New York in the 1960s (Jenner, 2011).^7^ In Disneyland, California, for example, sweet candy smells are diffused throughout the site. However, in this case, it is worth noting that the scent may well be used as much to boost food and beverage sales as anything else (see Spence, 2015a, for a review of olfactory food marketing). Meanwhile, at Disneyland’s California Adventure theme park, a gentle smell of citrus is apparently spritzed on visitors during a ride where they seemingly soar over a grove of orange trees (Legro, 2013). In this case, it is worth noting how the scent is semantically congruent with the scenery. In the setting of the theme park, as in many museum displays, the visitors get to smell what they see (or, more likely, a synthetic reproduction of it).

**Figure 2.**
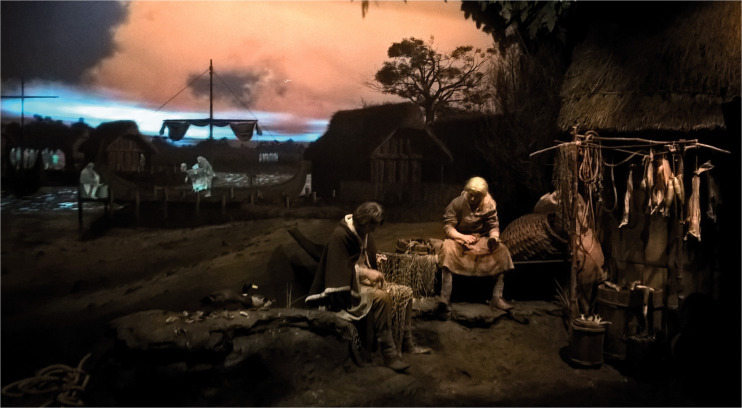
One of the Displays at the Jorvik Viking Centre Where a Different Scent Is Used to Augment Each of the Seven Tableaux (by Chemical Engineer – Own Work, CC BY-SA 4.0, https://commons.wikimedia.org/w/index.php?curid=58524884).

At the Jorvik Viking museum in York (UK), scent is used to help try and give the visitors a multisensory impression of what life would have been like for the inhabitants of York in the 10th century (AD 948) as they move through the various museum displays (see Figure 2). The smells include “burnt wood,” “rubbish acrid,”^8^ “fish market,” “beef,” “earth,” “rope/tar,” and “apples.” In this case, the problem of the various smells mixing is minimized by the fact that the visitors are whisked in a trolley through the various tableaux presented in different rooms.

Aggleton and Waskett (1999) conducted a study in which people’s long-term memory of the Jorvik Viking Centre was tested. These researchers found that just one whiff of the seven odours that had been used in the museum displays was sufficient to bring back memories of a previous visit to the museum, even for those whose had not visited for several years. The participants in this particular study were given a questionnaire about the visually observable features of the exhibits on display. While these results undoubtedly do highlight the fact that a visitor’s memories of a museum can be enhanced by the presentation of the appropriate scents (rather than unrelated scents or no scents at all),^9^ it is worth bearing in mind that this is typically not something that is going to be available to most museum visitors. Hence, from a curatorial standpoint, it would perhaps have been more interesting to know whether people’s memory for the details of an exhibit is better if it is scented than if, as is normally the case, there is no scent present (cf. Lwin & Morrin, 2012; Tomono et al., 2011).

One further point to note here is that it tends to be the unpleasant (rather than the pleasant or neutral) scents that increase a person’s immersion, or engagement, in an experience, at least in the context of virtual reality (e.g., Baus & Bouchard, 2017). It would therefore be interesting to determine whether it was the highly unpleasant odours (remember the “acrid rubbish” smell) that had more of an effect on the visitor’s memory of the Jorvik Viking Centre tableaux than the more neutral or positive scents.

Elsewhere, in a conference abstract, Knasko (1993) from the Monell Chemical Senses Center in Philadelphia reported how the presence of a congruent scent made people linger for longer at an exhibit in an anthropology museum (see Table 1 for a summary of studies). There were four scent conditions in Knasko’s study: no scent, incense (rated as pleasant and congruent with the display), bubble gum (rated as pleasant but incongruent with the display), and leather (rated as unpleasant but congruent with the display). The exhibit was scented for 4 days a week for a period of 8 weeks. Those in the incense group reported that they had learnt more than those quizzed after experiencing the odourless exhibit. The incense group also reported that the ambient odour had had a more positive influence on their enjoyment of the exhibit when compared with those exposed to the bubble gum or leather scent. One additional result to emerge from this study was that those exposed to the bubble gum scent reported being in a better mood than those in the no odour condition.

**Table 1.** Chronological Summary of Laboratory-Based Crossmodal Studies That Have Investigated the Impact of Scent on Participants’ Ratings of Visual Stimuli (Reproductions of Paintings and Photos), As Well As a Couple of Studies of Scent on People’s Response to Museum Exhibits.

Study	Number of participants	Were scents explicitly paired with pictures?	Olfactory stimuli	Results
Rotton (1983)	48	No, ambient scent	Very –ve	–ve scent lowered participants’ ratings of 4 paintings
Lorig & Thompson (1989)	93	No, ambient scent	Hedonically +ve	+ve scent of vanilla lowered women’s ratings of random line art
Herz & Cupchik (1993)	48	Yes	Hedonically –ve and +ve	Hedonic tone of scent affected hedonic rating of associated pictures
Knasko (1993)	–	Semantically congruent and incongruent scents	Hedonically +ve, –ve, and no scent	Hedonically +ve and semantically congruent scent led to increased learning and enjoyment
Knasko (1995)	90	No, ambient scent	Hedonically +ve and –ve	+ve scents led to increase in looking time and enhanced mood
Banks et al. (2012)	16	Yes	Hedonically –ve, +ve, and no scent	–ve scent lowered ratings of +ve and neutral IAPS pictures
Cirrincione et al. (2014)	86	No, ambient scent	Hedonically +ve and neutral scent	+ve scent reduced ratings of paintings and impaired participants’ memory
Vega-Gómez et al. (2020)	234	Yes, one scent in each of 3 rooms in museum	3 semantically congruent scents	Those visiting scented rooms rated the experience better and were more likely to return

*Note*. IAPS = International Affective Picture System.

More recently, Vega-Gómez et al. (2020) scented three rooms in a small regional Spanish museum dedicated to everyday life in the 19th and 20th centuries. In this case, the scents were presented at a low level and had been chosen to match the theme of the rooms, namely, a “clean clothes scent” for a bourgeois-class dressing room, the smell of “apple pie” for a working class kitchen, and the “smell of aftershave” for a barber shop. One hundred and two museum visitors were quizzed in the no scent condition, while a further 132 visitors were surveyed about their experience after having experienced the scented rooms. Intriguingly, the visitors rated the experience more highly when there was a scent present in the museum. What is more, the visitors reported that they would be more likely to return after having been exposed to the scented than the unscented displays (see Burton et al., 2009, on the importance of repeat custom in the museum sector). At the same time, however, it should also be born in mind that when scent is suddenly introduced, into a normally unscented setting, there is always a danger that any effects may reflect some sort of placebo effect (Pursey & Lomas, 2018, p. 365).

### Ambient Versus Targeted Odour Delivery

Before its closure, Vinopolis, the museum dedicated to wine in Southwark, London (1999–2015; e.g., Atkin, 2015), used scent displays in certain of its exhibits. Interested visitors were, for instance, able to put their nostrils to a line of nosepieces mounted in the wall to smell the distinctive bouquets of different wines. This represents one solution to the targeted delivery of scent (i.e., rather than trying to scent an entire space). Meanwhile, at the 2010 exhibition “How Wine Became Modern” at the San Francisco Museum of Modern Art, visitors were able to inhale the scents of wine from open carafes instead (Kino, 2012). Individual flasks with hand pumps to deliver a small burst of fragrance were also used in the *Something in the Air*—*Scent in Art* exhibition as the Villa Rot, in Germany (see Verbeek, 2018, p. 204).

A number of technical solutions to the problem of targeted scent delivery in front of a display have emerged in recent years (Lipps, 2018; Spence et al., 2017). One intriguing approach that has already been incorporated into several of the exhibits that have been mentioned so far in this review involves the use of movement-triggered scent release to release a small amount of scent when a visitor approaches an exhibit (see Kino, 2012; Pursey & Lomas, 2018). Devices such as the AromaShooter by Aromajoin are capable of projecting a directed pulse of odour to a specific location in front of a display (Obrist et al., 2017; Spence et al., 2017). Obviously, the more the delivery of the odourant can be targeted both spatially and temporally, the less of an odourant needs to be released in a space, hence reducing the danger of scent overload.^10^

Of course, *scent-sory* overload is precisely what certain olfactory artists would appear to have in mind. For example, just take the *Fear I, 2005*, exhibit by the Norwegian smell artist Sissel Tolaas, which consists of a room in which the synthesized smell of human sweat had been microencapsulated and embedded in white paint of gallery walls (Arning, 2006). Tolaas did much the same in another of her striking olfactory exhibits entitled “FEAR of smell—the smell of FEAR,” presented at the 2005 Tirana Biennale, where she synthesized the body smells of 15 men who were afraid of something, and once again microencapsulated them and mixed them in the white wall paint applied to the gallery walls. Those who have experienced the olfactory onslaught do not soon forget the experience (Howes, 2015). With her performance piece *Actual Odor* (1997), executed at the reception of Arizona State University Art Museum exhibition *Token City*, Angela Ellsworth also challenged olfactory sensibilities while playing with the notion of incongruency. According to Bacci (2015, p. 131), “The artist, elegantly made-up, wore a urine-soaked dress, whose stench people could clearly perceive but could not connect to her, since her sophisticated appearance defeated the expectation of uncleanliness” (see Wetzel & Müller-Alsbach, 2015, for a number of other highly invasive olfactory installations).

A number of other olfactory artists, such as Peter de Cupere, have also been prolifically creating olfactory/multisensory artworks (de Cupere, 2017). The Brazilian artist Ernesto Neto scented out the St. Louis Art Museum in 2000 as part of the Wonderland exhibition by using nylon stocks filled with spices such as cumin, cloves, and turmeric (see Shiner & Kriskovets, 2007, on Neto’s work *Mentre niente accade/While nothing happens* 2008, and other fragrant examples). And going one stage further, The Basel Museum für Gestaltung staged an exhibit in 1996 called “Aroma, Aroma” where some of smells were pumped out onto the street around the museum (Bendix, 2011). At the same time, it is worth remembering olfactory art historian Jim Drobnick’s (2014, p. 192) observation that “Since every space has some kind of scent, to some degree every olfactory artwork has to work with or against such residual odours.”

One of the challenges with working with ambient scent is that people have been shown to become rapidly anosmic to its presence should they happen to be attending to something else at the time. For instance, Forster and Spence (2018) demonstrated the existence of the phenomenon of “inattentional anosmia”—that is, a selective inability to smell an ambient odour (e.g., the aroma of coffee) if one has been performing an attention-demanding visual task. At present, the precise conditions under which those scents we are not aware of smelling can nevertheless still affect our judgements have not been thoroughly worked out. Though, what is clear is that even unconsciously perceived smells can sometimes still have a significant effect on us, and on our judgements (e.g., see Keller, 2014; Li et al., 2007).

One of the other challenges when considering the introduction of scent to the museum/gallery space is the possibly negative response that may be elicited in those suffering from multiple chemical sensitivities (e.g., Byatt, 2003; Fletcher, 2005). In fact, some who have been working to introduce scent as a regular element in the context of theatrical performance have suggested that the audience should perhaps be warned, in advance, of any performance, that smells will be used (McGinley & McGinley, 2018, p. 225). Much the same suggestion has been made by Jim Drobnick (2010), who has himself curated a number of olfactory exhibitions. The voluntary nature of olfactory exposure in those cases where the visitor has to sniff a carafe or nozzle actively presumably helps to address such concerns. That said, the arrival of the recent pandemic means that such direct physical contact with the installation in public spaces will likely fall out of favour for the foreseeable future.

### Scent Exhibits

Finally here, it is worth noting that rather than merely using scent as a means of augmenting the visual experience when viewing the art, several recent exhibits have focused primarily on scent itself have also been mounted. Stevenson (2014) mentions the existence of at least 10 museums around the world that are wholly devoted to smell, typically related with perfume and/or food and drink. He also notes the many scent-related exhibitions have been put on, over the years including *Adventures in Scent* at the British Museum in London in 2011. In addition, *The Art of Scent 1889–2012* exhibition took place at the Museum of Arts and Design in New York at the end of 2012 (Kino, 2012; Nieuwhof, 2017), while another major exhibition entitled *Belle Haleine*—*The scent of art* was put on at Museum Tinguely, Basel, Switzerland in 2015 (see Museum Tinguely, Basel, 2015). *Perfume: A Sensory Journey Through Contemporary Scent* was mounted at Somerset House in London in 2017 (Moore, 2017). In such cases, the visual displays tend to augment the olfactory experience rather than *vice versa.*

Some years ago, one German design student also documented a research project in which they had attempted to capture a range of scents by means of a series of abstract visual designs that were then mounted in an exhibition space (Langner, 1997). Although beyond the scope of the present article, it is worth noting that there has been what some have wanted to describe as an “olfactory turn” in the visual arts (Drobnick, 1998; Nieuwhof, 2017), with many artists starting to incorporate more of an olfactory element into their work than was previously the case (see Drobnick, 2005, 2014; Keller, 2014, for a number of such examples). At the same time, however, there has also been a concerted push to make the art of visually impaired artists more accessible to the general public as well (Salman, 2011), and this has sometimes incorporated an olfactory element.

### Interim Summary

The various examples reported in this section hopefully give an idea of how the sense of smell is coming to play a small, but increasingly important, role in the setting of the gallery/museum. It is, however, worth pointing out the distinction between the approach that one tends to see in the museum sector, where the semantically matching scent for an exhibit is used, and the approach that is typically found in art galleries where, instead, the scent would appear to be congruent with, but not necessarily exactly linked to, what one is seeing.^11^ The danger in the latter case, of course, is that the connection between the scent and the visual display may not be altogether clear.^12^ Hence, it may distract people from whatever they are viewing (cf. Gilbert, 2008; Parsons, 2009; Pursey & Lomas, 2018). And while the release of pleasant ambient odours may well help to increase approach behaviour and elevate people’s mood in the context of an exhibit (Knasko, 1995), it is worth remembering that it tends to be the unpleasant smells (remember the Viking acrid rubbish smell) that appear to have the most pronounced impact in terms of enhancing people’s immersion in a particular experience (e.g., Baus & Bouchard, 2017).

One of the criticisms that has sometimes been made of the use of scent in museum displays, in particular, concerns the use of synthetic rather than authentic odours (Keller, 2014). At the same time, it would seem likely that our response to the smells of the past is going to be very different from that for those whom it formed the olfactory backdrop to their everyday lives, and hence while the smell may well be similar, the responses is likely to be very different. This has led some commentators to question the appropriateness of introducing scent in the first place (see Eve, 2018; Morley, 2014, p. 119; Walsh, 1990, p. 287). As Kevin Walsh (1992, pp. 112–113) puts it:

The decontextualization of smells from an historical period and placement in a twentieth century tourist attraction seems highly dubious as each person visiting the centre will have a different perception or attitude towards a smell and it is quite likely that it will be very different from those held by the people who originally produced and lived with the smells. The problem is compounded by the fact that one begins to wonder if Victorian streets, Medieval universities and Viking villages were all steeped in the same, obviously-artificial, chemical-odor version of wet cats.

Of course, in the case of many ancient or historic smells, it is likely going to be necessary to make an educated guess as to what they once would have been like (e.g., think about recreating the smell of dinosaur dung; see Jenner, 2011). Odours are, after all, rarely preserved in the same way that artifacts from the other senses are (see Stevenson, 2014).^13^ At the same time, however, it is also worth highlighting the fact that the real power of unpleasant odours may sometimes derive from the fact that they are genuine, not synthesized. As an extreme example, just take Davis’ (1995, pp. 35–36) description of a visit to the pungent “Shoe Exhibit” at the Holocaust Museum in Washington, DC: “intellectual horror at an already familiar narrative of recent genocide is compounded by olfactory confirmation in a gallery where the odor of decomposing shoe leather cannot be obliterated by even the most energetic air conditioner.” She continues that “… the shoes’ odor invades visitors’ bodies and in so doing cements concepts to experience” (see also Bacci, 2015).

## Olfactory Modulation of Art: Laboratory Research

Over the years, a number of researchers have attempted to investigate the effect of various ambient odours, both positive and negative, and either congruent with, or unrelated to, the visual stimuli, that people are asked to evaluate in the setting of the laboratory. For example, in what is perhaps the first study of its kind, Rotton (1983) reported that people (*N* = 24 male and 24 female students) judged colour reproductions of four paintings (*Black Lines* by Kandinsky, *The Studio* by Picasso, *Flemish Proverbs* and *The Fall of Icarus* both by Brugel the Elder) to be significantly less professional, significantly less worthy, but no less tasteful when viewed in the presence of an unpleasant odour than when evaluating the same paintings in the absence of any ambient smell (though see Knasko, 1995). Note that the participants in this particular between-subjects study had to rate the paintings on 15-point scales anchored by extreme liking/disliking, amateur-professional, and worthless-priceless. The participants also rated photos of their peers and persons described by adjectives. The volatile chemical used in this case was ethyl mercaptan, described as smelling of rotting cabbage or sewer gas, though when presented in its pure form (as in Rotton’s study), it is apparently even more unpleasant than it sounds. Hence, a highly aversive olfactory stimulus was used, with no meaningful connection to the art.

In another between-subjects study involving pleasant scent, Lorig and Thompson (1989; as described in Lorig, 1992) had their participants evaluate examples of randomly generated computer line art (that had been preselected to be neutral) in a theatre. In this case, the room in which the participants rated the art was scented with either lavender or vanilla, released from a vial and diffused by a fan. The female, but not the male, participants rated the art as less attractive when they were exposed to the smell of vanilla, with the negative effect on ratings increasing with the length of the women’s exposure to the scent. Intriguingly, this result was obtained despite the fact that only three out of the 93 participants reported having noticed the scent when debriefed after the experiment.

Herz and Cupchik (1993) reported that women’s and, to a lesser extent, men’s aesthetic evaluations of paintings were intensified when the latter were presented with a matching (or congruent) odour. In this case, the participants were explicitly instructed to try and combine the experience of looking at the painting with the experience of the odour. Once again, 24 male and 24 female students acted as participants. Six positive, 6 negative, and 12 hedonically neutral paintings were displayed, each paired with one of 24 odours (12 hedonically pleasant and 12 hedonically negative). At the start of each trial, the participants had to rate the pleasantness, familiarity, and intensity of the odour on 7-point semantic differential scales. They then had to rate the artistic quality and visual complexity of the paintings that were displayed by means of a slide projector. The participants also responded to scales designed to assess the personal meaning and relevance of each work of art.

The results of this intriguing study revealed that the hedonic tone associated with the scent affected the participants’ ratings of the hedonic quality of painting. For example, the pleasantness of positively toned pictures, such as Renoir’s (1881) “Luncheon of the boating party,” was found to be intensified when the picture was paired with pleasant smells such as rose, pine, or jasmine when compared with the participants’ reactions that were reported when viewing the pictures in the presence of an unpleasant odour, such as fried onion, creosote, or rancid butter. The more extreme the emotional intensity evoked by the picture, the less influence the contextual smell had. There are a couple of unique features about this study that are worth noting: First, this is one of the only laboratory studies in which the participants were instructed to try and combine their response to the olfactory and visual stimuli. What is more, and in contrast to the scents used in museum displays (reviewed earlier), while the smells might well sometimes be hedonically congruent with the mood of the paintings, they were pretty much guaranteed to be semantically incongruent with it. It is worth bearing in mind that the attention of Herz and Cupchik’s (1993) participants would presumably have been drawn to the hedonic rather than the semantic associations of the scents by the various rating scales that they were asked to complete on first being exposed to each of the scents. This particular aspect of the experimental design may thus help to explain why it is that this study is one of the only ones where the crossmodal influence of scent on ratings of the paintings appeared to be influenced by the hedonic congruency between the component stimuli.

Knasko (1995) presented the participants (*N* = 90) in her between-subjects design with one of two odours, either a chocolate scent or a baby powder scent, with matching or mismatching pictures of foods containing chocolate (*N* = 6) or photos of babies (*N* = 6). There were also 12 neutral images. The participants viewed the slides in their own time. However, the results revealed little evidence that such semantic congruency had any significant effect on participants’ responses to the pictures. Pleasant scents led to longer looking time, and better mood. There was, however, no effect of congruency on participants’ responses.

More recently, Banks et al. (2012) presented pleasant (bergamot and muguet), neutral (air), and unpleasant odours (isovaleric acid and pyridine) while presenting pictures from the International Affective Picture System—specifically, three pleasant, three neutral, and three unpleasant affectively laden scenes. The participants (*N* = 16) in this pilot study were required to rate the images using a self-assessment mannequin. The participants were presented with 36 trials where the nine images were presented with each of the four odourants. The pictures were rated higher in the presence of one of the pleasant odours than in the presence of one of the unpleasant odours. The neutral air control condition was also rated higher than the unpleasant odour. Participants’ responses to positive and neutral images were reduced in the presence of a negative smell, while there was no effect of odour on ratings of the negative images. An enhanced electrodermal response was also reported in participants when exposed to a negative odour together with a negative image, when compared with when it was combined with a positive odour instead. Overall, therefore, Banks et al.’s results demonstrate that while the introduction of a negative scent can lower people’s ratings of images, the release of one of two positive scents did not raise their ratings above the no scent baseline condition. As such, these results would seemingly provide little encouragement for those wanting to use scent to enhance the visitor experience in the context of the art gallery.

Similarly, Cirrincione et al. (2014) have also reported that smelling a pleasant fragrance while viewing a work of art is not necessarily always a good idea, at least not if one wants people to remember what they have seen. The researchers in question assessed the impact of releasing one of two ambient scents on people’s perception and memory of a series of paintings by Russian-American abstract artist Mark Rothko and the Italian Arcimboldo (the latter famous for painting portraits of people composed of pieces of fruit and vegetable). A pretest revealed that a citrus scent was rated as somewhat more congruent with Arcimboldo’s work, while a sweet talcum scent was perceived to be a little more congruent with Rothko’s ouvre instead.^14^

The 86 undergraduates who took part in Cirrincione et al.’s (2014) main study were shown 15 works from each artist, presented on a computer monitor, in a random order. The participants were instructed to look at the pictures sequentially, in their own time, as if they were viewing them in an art gallery. One group of participants was exposed to the citrus scent, while the other group was exposed to the talcum scent. The participants evaluated each painting on arousal (from calming to exciting) and valence (from negative to positive) on 7-point scales. The participants also had to rate their liking of the works (from not at all to very much). After having viewed (and rated) the various works of art, the participants were then given a surprise memory test, in which the original 30 paintings were presented once again. This time, though, they were mixed-up with 30 additional paintings from the two artists that were used as “lures” or foils. The participants had to try and recognize which of the works of art they had seen previously. Surprisingly, however, the results showed that the presence of the sweet talcum scent lowered people’s evaluation of the art (relative to the more neutral citrus scent) while, at the same time, impairing their memory for what they had just seen. What is more, Cirrincione et al.’s results also suggested that the paintings were judged as being more arousing when they were viewed in the presence of the putatively incongruent scent.

One should, though, perhaps be careful about interpreting the results of this study, given the between-subjects nature of the scent manipulation involved. That is, different groups of participants were exposed to each scent, meaning that it is difficult to unequivocally rule out the possibility that the effects reported simply reflect baseline (individual) differences between the two groups of participants in terms of their response to the art (i.e., rather than being a result of the scent manipulation). Note, though, that several of the other studies reported in this section (e.g., Lorig & Thompson, 1989; Rotton, 1983) are also open to the same criticism. In addition, given that there was not a baseline no scent condition in Cirrincione et al.’s (2014) study, it is impossible to say, in hindsight, whether the mere presence of ambient scent in their study may have influenced people’s experience (i.e., regardless of its degree of match, or congruency, with the paintings concerned).

### Interim Summary

The most parsimonious conclusion to draw from the limited research that has been published to date on the effects of ambient scent on people’s responses to pictures or works of art (as studied in the laboratory) would appear to be that trying to enhance experiences by means of the addition of ambient scent can, counterintuitively, actually hinder their evaluation of the art when viewing it, not to mention their memory of the art thereafter. No matter whether the ambient scent is pleasant or unpleasant, the impact on participants’ ratings is, more often than not, negative. At the same time, however, it should also be noted that none of the studies that have just been reviewed in this section (e.g., Banks et al., 2012; Cirrincione et al., 2014; Herz & Cupchik, 1993; Lorig & Thompson, 1989; Rotton, 1983) had particularly high ecological validity—all being conducted in the setting of the science lab while participants briefly viewed reproductions of the artworks. What should also be stressed is that all of the participants were undergraduates—and all were from Western, educated, industrialized, rich, and democratic (or WEIRD for short) societies (see Henrich et al., 2010). As such, one presumably needs to be cautious about generalizing from this very restricted demographic to the population at large (see also Arnett, 2008). What also emerges from the laboratory studies that have been conducted to date is that, if anything, women would appear to be more influenced than men by the presence of ambient scent (see Herz & Cupchik, 1993; Lorig & Thompson, 1989).

One of the reasons why the ambient scent in many of the studies reported in this section may not have had more of an effect on participants’ visual ratings is that in only one study, namely Herz and Cupchik (1993), were the participants actually given any reason to link the scent with whatever they happened to be looking at. Given that the same scent was often presented with a number of different works of art (e.g., in the studies reported by Cirrincione et al., 2014; Lorig & Thompson, 1989; Rotton, 1983), the participants would presumably have had little reason to link, integrate, or perceptually group, what they were viewing with what they were smelling (see Spence, 2015b, on the notion of crossmodal perceptual grouping). The one exception to this generalization comes from Herz and Cupchik’s study where the participants were actually instructed to integrate their feelings about the specific pairing of olfactory and visual stimuli (though, in this case, there was no semantic link between sight and scent). This contrasts, for example, with those more theatrical scented events where the link between the ambient scent and the visual stimuli has been made much more explicit to the audience (e.g., Legro, 2013; Shepherd-Barr, 1999). Certainly, the putative link between the scent and the art or museum display is likely to have been more obvious to those museum visitors described in the previous section.

## Explaining the Influence of Ambient Scent on the Perception of Art/Museum Displays

The various results reported in the last two sections highlight how people’s responses to both works of art and museum displays can be influenced by the presence of ambient scent. At the same time, however, the laboratory research also demonstrates that people’s response to paintings can be influenced by the presence of ambient scent. It is not, though, always so clear whether the relation between the scent and the work on display matters, and/or whether the link, or congruency, between the visual and olfactory stimuli was emphasized or not. In this section, I review a couple of the principal routes/mechanisms by which what people smell might come to influence what they say, or remember about the art or display.

### Sensation Transference

One popular explanation for why the presentation of contextual stimuli, be they olfactory or auditory, might influence what people have to say about whatever it is they are rating is “sensation transference.” The basic idea is that the more people (dis-)like an ambient scent, the more they will (dis-)like whatever it is that they happen to be viewing/rating at the time (see also van Reekum et al., 1999). For instance, Reinoso-Carvalho et al. (2019a) recently provided evidence of “sensation transference” from background music to people’s ratings of taste/flavour. Specifically, the more the participants liked the music they were listening to, the more they liked the beer that they had been given to taste. To the extent that sensation transference is one of the relevant mechanisms/explanations behind olfaction’s crossmodal influence on people’s visual ratings, the suggestion would be simply to consider choosing the most pleasant scent rather than necessarily worrying about the congruency between scent and sight (see Knasko, 1995). Problematically, though, for such a suggestion, it tends to be negatively valenced smells that tend to have more of an effect on people’s ratings than positive scents (cf. May & Hamilton, 1980).

At the same time, however, it is also important to remember that Parsons (2009) has cautioned against such an approach, at least in the context of a normally odourless retail setting. According to Parsons’ research, there needs to be at least some connection between the scent and type of store to increase the likelihood of the scent having a positive effect on the customers’ response. Or, as Gulas and Bloch (1995) put it, while the scent of flowers may be generally perceived as pleasant, it would be wholly inappropriate for a motorcycle shop. One might presumably make the same argument when considering the scenting of a motoring or railway museum. Semantic congruency is, in other words, likely to be important to determining the visitor response (see also Bacci, 2015, on the notion of scent congruency in the context of the multisensory museum).

### Attentional Modulation

One of the other ways in which those ambient scents that are closely linked to a particular colour may influence a viewer’s response to a work of art is by directing their attention preferentially to those regions of the scene whose colour happens to be similar to that of the scent they are smelling (e.g., Chen et al., 2013; Michael et al., 2003; Robinson et al., 2013, 2015; Seigneuric et al., 2010; Seo et al., 2010; Tomono et al., 2011). This suggestion is based on research showing that people’s eye movements do indeed tend to be biased towards those areas of a visual scene whose colour matches that associated with the scent (e.g., think only of how people will tend to look at red items when smelling a strawberry scent; see Wada et al., 2012).

Remember also how this was the idea behind the choice of scents for one of the works displayed at the *Tate Sensorium*. In particular, David Bomberg’s *In the Hold* (c. 1913–1914) was meant to be viewed while visitors sniffed one high-pitched scent chosen to bring out the blues in the painting, while the other scent was intended to bring out the browns and ochers instead. As has been noted already, though, it is unclear how successful Flying Object was in using the crossmodal correspondences between scent and hue (cf. Spence, in press). That said, evidence that retronasal olfactory/gustatory stimuli might also tend to draw a viewer’s attention to the crossmodally associated colour comes from reports by those who tasted the burnt cocoa nib dark chocolate with dust while viewing Francis Bacon’s Hyde Park painting at the *Tate Sensorium* and who said that it: “really made the black almost throb” (Pursey & Lomas, 2018, p. 365).

On the other side, however, when the ambient scent and the visual display do not match (i.e., when they are experienced as incongruent), there is a danger that the presence of scent may well distract, and/or distance, the viewer from what they are looking at (Pursey & Lomas, 2018; cf. Gilbert, 2008). At the same time, Cirrincione et al. (2014) have suggested that certain of their empirical results could best be explained in terms of the increased arousal someone viewing art may experience when in the presence of an incongruent scent. Their suggestion is that such crossmodal incongruency may be arousing and hence potentially lead to a change in a viewer’s evaluation of the art.^15^ Incongruency, note, was very much what the Surrealists were interested in delivering when, for example, they incorporated the powerful smell of roasting Brazilian coffee at the 1938 International Surrealist Exhibition held at the Galerie des Beaux Arts. At the same time, however, this smell was also semantically meaningful (Kachur, 2001). In particular, the smell may have been chosen both because of its incongruency with the interior setting of the gallery (at the time it would have been more congruent with an outdoor café), and also the fact that it was specifically (and to some, recognizably) the smell of Brazilian coffee roasting perhaps referencing 1938 as the year in which the Brazilians joined the Surrealist movement (Verbeek, 2015; Verbeek & van Campen, 2013).

Another version of the attentional account that has been suggested by David Lomas, from the Department of Art History, at the University of Manchester when writing about the multisensory interventions introduced by Flying Object at the *Tate Sensorium*, are that “though I can’t help thinking that some at least of the benefit reported can be put down to simply prolonging the duration of the encounter with the artwork” (Pursey & Lomas, 2018, p. 365). The suggestion here is that consuming the flavourful chocolate may result in the viewer simply paying more attention to the work and, by so doing, may enhance the richness of the viewer’s sensory experience. Future empirical research testing the suggestion that lingering time can be increased by the introduction of the appropriate ambient scent would undoubtedly be beneficial, moving forward (remember that this was one of the measures reported in Knasko’s, 1993, conference abstract, as well as in Knasko’s, 1995, laboratory study).

## Crossmodal Congruency: How Is It To Be Defined?

One issue that is undoubtedly worthy of further empirical consideration is how exactly crossmodal congruency should be defined when it comes to the pairing of scents with works of art or museum exhibits (see also Bacci, 2015, on this theme). Perhaps the most straightforward kind of congruency is semantic, as when scents are matched to the items on display. This was the kind of congruency that was assessed in the laboratory by Knasko (1995) when she presented odours (chocolate scent or baby powder scent) with matching or mismatching pictures of foods containing chocolate or photos of babies. As should have become apparent in this review, the semantic matching approach to scent appears to be much more common in the museum sector than in the art gallery. That said, Francesca Bacci (2015, p. 129) draws attention to the visual-olfactory semantic congruency that is at play in Jannis Kounellis’s *Untitled* (2001). She writes that

 … one will find visual confirmation that this work utilizes coffee, whose pungent odor pervades the air. The visual and olfactory sensations reinforce each other as one strolls through the space characterized by numerous rhymically organized columns of hanging welded steel trays filled with coffee grounds.

Over the years, various commentators have been minded to complain about the use of semantically congruent scents that are nevertheless synthetic or artificial smelling (e.g., Keller, 2014; Walsh, 1992). It should, though, be born in mind here though that when done well, synthetic scents can, in many cases, be impossible to distinguish from the real thing.

Others, meanwhile, would appear to have given up on the notion of semantic congruency and instead investigated the effect of introducing scents that are either hedonically congruent or incongruent with whatever happens to be displayed (Banks et al., 2012; Herz & Cupchik, 1993). Hedonic (in-)congruency has been what pretty much every laboratory study of the impact of scent on paintings has chosen to investigate. What is currently not so clear from the literature that has been published to date is when exactly, the hedonic congruency between odour and visual display matters, and when it does not.

One way in which to assess the emotional associations of paintings or fragrances is by means of the semantic differential technique (Osgood et al., 1957). According to the semantic differential approach, people are required to rate stimuli (e.g., paintings, perfumes, pieces of music, or even simple sensory stimuli, such as colours, or concepts) on a series of semantic differential scales anchored by pairs of adjectives such as good–bad, active–passive, and dominant–submissive (Dalton et al., 2008; Spence, 2020a).^16^ According to one popular suggestion, stimuli that are ranked similarly in terms of the three main dimensions (pleasure, arousal, and dominance) will be more likely to be rated as matching.

Another kind of congruency between scent and vision that has, on occasion, been investigated is in terms of crossmodal correspondence references the direct links between colour (or other aspects of visual stimulation) and scent (Langner, 1997; see Spence, in press, for a review). Note that this was precisely the kind of correspondence that was targeted by the two scents chosen to augment the experience of David Bomberg’s painting *In the Hold* as part of the multisensory *Tate Sensorium* experience (Pursey & Lomas, 2018). As mentioned earlier, though, while semantically meaningful scents have been shown to draw people’s attention to the colour of the reference object (e.g., Chen et al., 2013; Seigneuric et al., 2010; Seo et al., 2010), it is an open question as to whether more abstract scents (i.e., those that do not obviously reference a particular coloured source object) also draw people’s attention to the crossmodally corresponding colour in a scene as well. So, for example, I am aware of no evidence that abstract, scents such as those introduced to match the Bomberg painting at the *Tate Sensorium*, actually bias people’s eye movements, plausible though the underlying idea sounds (cf. Kuang & Zhang, 2014). Intriguingly, the famous perfumer Edmond Roudnitska was certainly convinced of the existence of crossmodal correspondences between visual and olfactory art (Stamelman, 2006, p. 186).

Finally here, it is important to recognize that crossmodal congruency might also occur in terms of what has been referred to as cross-media artistic styles (e.g., Hasenfus et al., 1983; see also Siefkes & Arielli, 2015). However, while this kind of crossmodal matching has been established in the case of matching paintings, music, poetry, and even architecture, scents/fragrances are not typically described in terms of an artistic style such as Impressionist, Abstract, or Baroque. That said, scattered through the literature, such claims have, on occasion been made by perfume experts. So, for example, the famous perfumer and theoretician, Edmond Roudnitska, once described Coty’s *l’Origan* perfume from 1905 as “‘a translation of fauvist sensibility’, borrowing all its ‘vividness, violence and audacity’ (Stamelman, 2006, p. 186)” (quoted in Verbeek, 2018, p. 202). The 12 fragrances chosen for The Art of Scent 1889–2012 exhibition in New York were described using terms such as Modernist, abstract, or Brutalist. Meanwhile, Chandler Burr who put the exhibition together talks about Oliver Cresp’s cotton-candy-scented Angel perfume from 1992 as “a work of beautiful overt Surrealism” (Kino, 2012).

Given the many different ways in which the congruency of scent and visual stimuli might be defined, further research is undoubtedly needed to determine the conditions under which each type of congruency is most salient and/or effective in terms of enhancing a visitor’s experience of a display at an art gallery or museum (see also Bacci, 2015).

## Conclusions

While many people have considered the effects of adding scent to art and museum exhibits,^17^ the results of the academic research would appear to suggest that the addition of this normally unstimulated sense will not necessarily enhance the multisensory experience of those who are exposed to it (cf. Elgammal et al., 2020, for a similar discussion of the costs and benefits of introducing digital technology into the museum setting). While the use of congruent scents have been shown to enhance people’s self-reported willingness to return to a museum (Vega-Gómez et al., 2020), there is also a danger that it may distract from the works on display (cf. Gilbert, 2008). And, regardless of any crossmodal effects of scent on a viewer’s experience of a work of art or museum display, there are also significant practical challenges around ensuring the appropriate distribution of scent in/through a space (Drobnick, 2014; Keller, 2014). While the challenges are less difficult than when trying to deliver and clear a sequence of smells, as in the setting of the cinema, say (see Gilbert, 2008), they are nevertheless nothing to be sniffed at (as it were).

Furthermore, analogous to the attempts to augment the experience of art by means of scent, it is worth noting that there is a much larger (and largely parallel) body of research that has attempted to investigate whether background music can also be used to augment/enhance people’s experience of visual works of art (see Parrott, 1982; Spence, 2020a, for a review).^18^ The introduction of a sonic backdrop to an arts/entertainment experience undoubtedly has the advantage of being easier to control (i.e., in terms of the diffusion of sound, for instance, using directional audio; Davis, 2015). At the same time, however, the available research has yet to provide a clear story concerning the consequences of augmenting visual art, primarily paintings, but also, on occasion, sculpture (see Bremner, 2019) by means of the deliberate stimulation of another sense, be it sound (Baumgartner et al., 2006; May & Hamilton, 1980) or scent.

As such, those considering whether to introduce scent into an art gallery or museum setting would do well to consider what outcome they hope to achieve, and whether it will necessarily deliver the positive effects that are hoped for, however they may be defined. For, as has been argued here, it is important to note that, as yet, it is simply not possible to predict what the outcome will be of adding an additional sensory stimulus (be it a scent or, as it happens, a soundscape/music excerpt), to an exhibition or display. What is more, in the case of ambient scent, one probably needs to think carefully about whether to try and make the scent congruent with the visual display, and if so, quite which sort(s) of crossmodal congruency to go for.

Finally, here, it is worth noting how when scents are chosen specifically to modify a viewer’s experience of a work of art, this raises the thorny question whether it is actually appropriate to intervene between the artist and their audience. David Lomas, from the Department of Art History, at the University of Manchester captures this point when writing about the *Tate Sensorium* exhibition that

To conclude, it is quite a complicated matter to unpick what’s going on with an intervention such as “Tate Sensorium,” and doing so is unlikely to change the minds of those who, on the one hand, believe that multisensory experience is ipso facto a “good thing,” and definitely superior to vision alone; and, on the other, those for whom interfering with a picture they see as complete in itself is a mild form of desecration. (Pursey & Lomas, 2018, p. 365)

This concern then perhaps also links to the more general question about the most appropriate context, or environment, in which to display works of art. Getting to the bottom of whether the cube in which so much of art is currently displayed should be white, and/or anosmic, is, though, a question for another day.

## Notes

1 For instance, the Melbourne Museum in Australia, the National Museum of Scotland in Edinburgh, and the Kelvingrove Museum and Art Gallery in Glasgow, not to mention the Jewish Museum in Berlin, have all tried to engage more than just their visitors’ visual sense in recent years (McIntyre, 2009). Meanwhile, according to Kaye (2004), ambient scents have been used to influence the atmosphere in Bow Street Old Whiskey Distillery in Dublin and the Natural History Museum in London. Jenner (2011) mentioned the scenting of the Trafalgar room of Madame Tussaus Waxworks in London.

2 Wetzel and Müller-Alsbach (2015) also draw attention to the pseudoscientific experiment that surrounded Carsten Höller and François Roche’s, *Hypothèse de grue*, 2013, a fog-filled darkened room.

3 This was, in fact, one of the challenges that drew architectural firm Diller Scofidio & Renfro to the redevelopment project associated with mounting *The Art of Scent 1889–2012* exhibition at the Museum of Arts and Design in New York at the end of 2012. As Liz Diller, a partner in the firm, puts it: “‘The real excitement and lure’ of designing the scent exhibition … was in figuring out ‘how to make this work in a place that privileges vision as the master sense’” (as quoted in Kino, 2012).

4 However, anyone hoping to get a whiff of *The Mona Lisa* would have been sorely disappointed, as no perfume was created for what is undoubtedly one of the Louvre’s most famous works of art.

5 Separately, though, it would have been interesting, from an experimental perspective, to see how successful the noses were in creating scents that members of the general public would have spontaneously matched to the appropriate work of art under forced choice conditions (cf. Spence, in press).

6 It is, though, unclear on what basis these putative colour–odour crossmodal correspondences were established (see Spence, in press, for a review).

7 Talking of how the scenting of New York galleries has changed over the decades, curator Caroline Jones (2006, p. 46) also suggests to “Compare the galleries of the Museum of Modern Art (as of its 1939 ‘International Style’ incarnation) with the 1913 Armory Show, where swags of evergreens would have perfumed the milling crowds.”

8 This a very pungent Viking toilet smell that apparently evokes disgust in many of those who experience it.

9 This attempt to recreate the past with smell fitting with Russian author Vladimir Nabokov’s (1970) comment that “Nothing revives the past so completely as a smell that was once associated with it” (p. 60).

10 When scenting open spaces, one also needs to take account of the staff who may well be exposed to any ambient fragrance to a much greater extent than is the occasional visitor (see Vega-Gómez et al., 2020, on this point).

11 As Tom Pursey, one of the people behind the multisensory *Tate Sensorium* exhibition, puts it: “If we had just some painted apples, and we gave someone an apple, That’d be really boring” (quoted in Volpicelli, 2015). The pleonastic use of scent to reproduce what is seen would appear to hold little appeal in the context of the art gallery, or so it would seem.

12 Relevant in this regard, in his olfactory installation at the Museum of Hunting and Nature in 2017, Antoine Lie announced only two of the four fragrances that had been introduced. Two of the museum’s rooms were fitted out and specially odourized for the occasion. At the same time, however, the designer-perfumer had also introduced a couple of other scents into another two of the rooms in the permanent collections, without the public being aware of which (*Le sentiment de la Licorne*, Musée de la Chasse et de la Nature [Paris], May, 2017; see Castel, 2018).

13 Although note that some ancient artefacts have been reported to retain some of their distinctive scent over millennia (e.g., Koloski-Ostrow, 2014).

14 To be absolutely clear, the participants in the pretest were asked to “rate the extent to which the scent and the painting are congruent, or consistent, with one another” (Cirrincione et al., 2014, p. 620).

15 Consistent with such a view, Eskine et al. (2012) have shown how the increased arousal elicited by watching a scary movie clip can lead to enhanced responses to the art that is rated immediately thereafter.

16 That said, it may be that it is precisely the more complex, or intricate, works of art that elicit differing responses from observers (Snitz et al., 2016).

17 While, at the same time, often eyeing-up the marketing opportunities associated with the sale of scented products in the museum or gallery gift shop (e.g., Bremner, 2019; Drobnick, 2014; Levent & Pascual-Leone, 2014).

18 In 2012, for instance, music by Velvet Underground was played in the *Regarding Warhol: Sixty Artists, Fifty Years* at the Metropolitan Museum of Art (Drobnick, 2014). Meanwhile, in 2015, The National Gallery paired audio interpretations with a number of the paintings as part of its Soundscapes exhibition (Davis, 2015).
